# Alcohol abstinence and mortality in a general population sample of adults in Germany: A cohort study

**DOI:** 10.1371/journal.pmed.1003819

**Published:** 2021-11-02

**Authors:** Ulrich John, Hans-Juergen Rumpf, Monika Hanke, Christian Meyer

**Affiliations:** 1 University Medicine Greifswald, Prevention Research and Social Medicine, Institute of Community Medicine, Greifswald, Germany; 2 German Center for Cardiovascular Research, partner site Greifswald, Greifswald, Germany; 3 University of Luebeck, Department of Psychiatry and Psychotherapy, Research Group S:TEP, Luebeck, Germany; Addis Ababa University / King’s College London, ETHIOPIA

## Abstract

**Background:**

Evidence suggests that people who abstain from alcohol have a higher mortality rate than those who drink low to moderate amounts. However, little is known about factors that might be causal for this finding. The objective was to analyze former alcohol or drug use disorders, risky drinking, tobacco smoking, and fair to poor health among persons who reported abstinence from alcohol drinking in the last 12 months before baseline in relation to total, cardiovascular, and cancer mortality 20 years later.

**Methods and findings:**

A sample of residents aged 18 to 64 years had been drawn at random among the general population in northern Germany and a standardized interview conducted in the years 1996 to 1997. The baseline assessment included 4,093 persons (70.2% of those who had been eligible). Vital status and death certificate data were retrieved in the years 2017 and 2018.

We found that among the alcohol-abstinent study participants at baseline (447), there were 405 (90.60%) former alcohol consumers. Of the abstainers, 322 (72.04%) had met one or more criteria for former alcohol or drug dependence or abuse, alcohol risky drinking, or had tried to cut down or to stop drinking, were daily smokers, or self-rated their health as fair to poor. Among the abstainers with one or more of these risk factors, 114 (35.40%) had an alcohol use disorder or risky alcohol consumption in their history. Another 161 (50.00%) did not have such an alcohol-related risk but were daily smokers. The 322 alcohol-abstinent study participants with one or more of the risk factors had a shorter time to death than those with low to moderate alcohol consumption. The Cox proportional hazard ratio (HR) was 2.44 (95% confidence interval (CI), 1.68 to 3.56) for persons who had one or more criteria for an alcohol or drug use disorder fulfilled in their history and after adjustment for age and sex. The 125 alcohol-abstinent persons without these risk factors (27.96% of the abstainers) did not show a statistically significant difference from low to moderate alcohol consumers in total, cardiovascular, and cancer mortality. Those who had stayed alcohol abstinent throughout their life before (42; 9.40% of the alcohol-abstinent study participants at baseline) had an HR 1.64 (CI 0.72 to 3.77) compared to low to moderate alcohol consumers after adjustment for age, sex, and tobacco smoking. Main limitations of this study include its reliance on self-reported data at baseline and the fact that only tobacco smoking was analyzed as a risky behavior alongside alcohol consumption.

**Conclusions:**

The majority of the alcohol abstainers at baseline were former alcohol consumers and had risk factors that increased the likelihood of early death. Former alcohol use disorders, risky alcohol drinking, ever having smoked tobacco daily, and fair to poor health were associated with early death among alcohol abstainers. Those without an obvious history of these risk factors had a life expectancy similar to that of low to moderate alcohol consumers. The findings speak against recommendations to drink alcohol for health reasons.

## Abstract 2

In this cohort study conducted over 20 years, Ulrich John and colleagues examine the relationship between alcohol abstinence and mortality in a German adult population.

## Author summary

**Why was this study done?:**

- According to evidence, alcohol abstinence seems to be associated with a lower life expectancy than low to moderate alcohol consumption.
- Individuals who abstain from alcohol might include those who have risk factors that can be reasons for the shorter life expectancy compared to low to moderate alcohol consumers.

**What did the researchers do and find?:**

- A random adult general population sample had been drawn and the study participants been inquired about their consumption of or abstinence from alcohol in a first contact.
- Twenty years later, proportions of deceased and time to death were determined and compared between former alcohol abstainers and low to moderate alcohol consumers among the 4,028 study participants.
- The findings show that 91% of the alcohol abstainers at first contact had consumed alcohol in life before and 72% had a history of one or more criteria of a former alcohol or drug use disorder, former risky alcohol consumption, efforts to cut down or to stop drinking, daily tobacco smoking, or fair to poor health.
- In terms of mortality rate, no statistically significant difference was apparent between alcohol abstinent persons without these risk factors and who had self-rated their health as good to excellent and persons who consumed low to moderate amounts of alcohol.

**What do these findings mean?:**

- Former alcohol use disorders, risky consumption, efforts to cut down on drinking, ever daily tobacco smoking, and fair to poor health may predict early death among alcohol abstainers.
- Healthy alcohol abstainers who have no alcohol- or tobacco-related risk factors may not have a higher mortality than low to moderate alcohol consumers.
- The findings speak against recommendations to drink alcohol for health reasons.

## Introduction

Growing evidence speaks against the J-shaped curve according to which not only heavy drinkers but also alcohol abstainers have a higher total, cardiovascular, and cancer mortality than low to moderate drinkers [1–4]. According to the Global Burden of Disease study, moderate alcohol consumption was not protective against mortality from alcohol-related disorders [5]. Meta-analysis data revealed that persons who had abstained from alcohol their entire life before had no higher risk of all-cause or coronary heart disease mortality than low to moderate drinkers (<25 grams alcohol per day) [4,6]. It has been concluded that it is safest not to drink alcohol [5]. Second, for cancer of the female breast, even low drinking amounts (<7.5 grams pure alcohol per drinking day) were associated with higher risk of death compared to abstainers [7]. No evidence for a low-risk threshold of alcohol for breast cancer has been found [8].

Concern has been raised over potential causes of increased mortality risk among study participants who affirmed not to drink alcohol [4,9,10]. After limiting abstainers to those who indicated that they have been abstinent their entire life before, no increased mortality risk was found compared to low-volume alcohol consumers [4]. However, to choose lifetime abstainers as a comparison group might not be sufficient in the search for evidence on potential causes of higher mortality rates among alcohol abstainers. Both lifelong abstainers and former drinkers may include subpopulations with a variety of risk factors that may contribute to death. These are insufficiently known. They include former alcohol or drug use disorders, particularly dependence or abuse, risky alcohol drinking, tobacco smoking, and fair to poor health in general. Among alcohol-abstinent subgroups in the general population, individuals exist who have stopped drinking due to an alcohol or drug use disorder. However, little is known about how large this group is. There is a lack of studies about alcohol drinking or tobacco smoking histories of subgroups among those in the general adult population who indicated that they currently live abstinently from alcohol. Current alcohol abstainers include former drinkers and lifetime abstainers. However, lifetime abstainers have been revealed by cohort data to be a small minority. Only 1.7% did not have consumed any alcohol throughout their life until age 50 or higher in a cohort followed for more than 30 years [11]. Tobacco smoking has a high mortality risk itself but may also add to combined effects of alcohol and tobacco on mortality risk [12]. Particularly high risks of death have been revealed by data from use of alcohol and tobacco [13,14]. Combined effects of former alcohol consumption and tobacco smoking on disease and death may be supramultiplicative [12]. Cohort study data revealed that after controlling for smoking status, alcohol abstainers did not have higher total or cardiovascular mortality rates than low to moderate alcohol consumers [4,6]. Smokers did not seem to have any mortality benefit if they consumed low to moderate amounts of alcohol [15]. Further health conditions have been investigated by the assessment of self-rated health, which turned out to be associated with mortality. Increased risks of people who disclosed fair or poor health in their own view have been shown for total [16], cardiovascular [16,17], and cancer mortality [16], in contrast to those with good, very good, or excellent health.

The purpose of the present paper was to analyze risk factors for early death among adult respondents who indicated that they had abstained from alcohol during the last 12 months prior to an interview at baseline. The risk factors included former alcohol or drug dependence or abuse, alcohol risky drinking, having tried to cut down or to stop drinking, tobacco smoking, and self-rated fair to poor health. Twenty years later, a mortality follow-up was conducted.

## Methods

### Sample

In a northern German region, a random adult population sample aged 18 to 64 years was drawn in 1995 using the registration office data in which every resident has to be enlisted by law. The area had been chosen by sociostructural criteria. It comprised the city of Lübeck and 46 surrounding municipalities. This study area covered a total of 193,452 residents at age 18 to 64 [18]. Among the 5,829 individuals eligible for the baseline study, 4,093 (70.2%) interviews had been completed July 1996 to March 1997, and 4,075 were analyzed [18].

A mortality follow-up was conducted from April 2017 to April 2018. The median number of years from the baseline interview to the ascertainment of vital status was 20.6 years [19]. Vital status was ascertained for the 4,075 baseline study participants. Vital statistics data were retrieved from the residents’ registration files at the place of the last residence. In a first step, data of the residents’ registration office that had been responsible at the time of the baseline interview were retrieved. Second, when address changes had been documented, we received data of the registration offices in the single communities. The last place of residence was provided and the information whether the individual was alive or at which date deceased. Residents’ registration offices in the respective federal states of Germany and in single places of residence in other nations were contacted. Among the 4,075 study participants with complete baseline data, for 47 persons, vital statistics data could not be proved. For 4,028 (98.8% of 4,075), study participants’ vital status was ascertained. This is our final sample for the reported data analysis.

### Assessments

#### Baseline

Alcohol abstinence and consumption during the last 12 months prior to the interview were assessed by the Alcohol Use Disorder Identification Test-Consumption (AUDIT-C) questions [20], because they are standardized, well known, easy to administer [21], and turned out to predict mortality [1]. The AUDIT-C includes frequency and quantity of alcohol drinking: (1) How often did you have an alcoholic drink in the past 12 months? (0: never, 1: once a month or less, 2: 2 to 4 times a month, 3: 2 to 3 times a week, 4: 4 times a week or more often); (2) If you have an alcoholic drink, how many glasses do you typically drink at 1 day? (a small glass or a bottle of beer, a small glass of wine or sparkling wine, spirits, or liquor) (0: 1 to 2, 1: 3 to 4, 2: 5 to 6, 3: 7 to 9, 4: 10 or more); (3) How often did you drink 6 or more glasses in a row? (0: never, 1: less than once a month, 2: once a month, 3: once a week, 4: daily or almost daily). The 3 items are summed up to the AUDIT-C sum score. Its empirical range was 0 to 12. We grouped the AUDIT-C sum score into abstinence (0), low to moderate (1 to 3), moderate to high (4), high (5), very high (6 to 7), and extremely high alcohol consumption (8 to 12). Lifetime abstinence from alcohol consumption was part of the interview. Lifetime abstainers were those who answered “No” to the question whether they had ever in life drunk a glass of an alcoholic beverage.

The baseline interview included risk factors that speak against a recommendation to drink alcohol: one or more criteria for lifetime alcohol or drug dependence or abuse fulfilled, alcohol risky drinking, having made efforts to cut down or to stop drinking, tobacco smoking status, and self-rated health. The criteria for alcohol dependence, alcohol abuse, drug dependence, and drug abuse had been assessed using the German version of the Composite International Diagnostic Interview (M-CIDI) [18,22]. It provides a diagnosis of alcohol dependence, alcohol abuse, drug dependence, and drug abuse during lifetime according to the Diagnostic and Statistical Manual of Mental Disorders, fourth edition, of the American Psychiatric Association [23]. Alcohol risky drinking was assessed on grounds of questions as part of the alcohol dependence diagnosis and was estimated to be present if men said they consumed 40 or more and women to consume 20 or more grams pure alcohol per day [18,24] over a time span of 6 or more months in their life. Typical alcohol consumption in life before baseline was calculated using the number of single drinks on a typical drinking day. An illustration of glasses and bottles of single alcohol beverages including the number of units of pure alcohol was presented [25]. One unit was equivalent to 9 grams pure alcohol or, e.g., 0.25 liter of beer or 0.1 liter of wine. In addition to the quantity, the frequency of alcohol consumption per week or month was assessed. Those who consumed alcohol 3 or more times per week or 30 or more grams pure alcohol per day were asked whether they had made efforts to cut down or stop alcohol drinking using a standardized question (“I try to cut down on drinking alcohol or to stop drinking alcohol at all.”). The answer scale included 5 ranks from “not at all” to “absolutely.” Tobacco smoking status included never smokers, ever less than daily smokers, former, and current daily smokers. Never smokers were those who indicated that they never had smoked tobacco. Ever less than daily smokers reported a history of smoking over a time period of 4 or more weeks but not daily. Former smokers were smokers who had disclosed daily tobacco smoking in life before over a time period of 4 or more weeks but not during the last 12 months prior to the baseline interview. Current smokers had smoked daily during the last 12 months prior to the interview. They were classified into those with 19 or less and those with 20 or more cigarettes per day during the time in their life when they smoked the highest number of cigarettes per day. To estimate self-rated health, we used the interview question, “How would you rate your health in general?” The answer categories were “excellent,” “very good,” “good,” “fair,” and “poor” [26] translated into German language.

#### Mortality follow-up

Mortality was calculated as total, cardiovascular, and cancer mortality. For cardiovascular and for cancer mortality, we used the death certificate information. Based on the information of the residents’ registration office about the date of death, we retrieved the death certificates from the local health authorities at the place of residence of the individual. The death certificates included health disorders that inferred death, which were a main causal or which were a contributing disorder in death. In total, a maximum of 15 disorders and 11 disorders as found by autopsy could be given by the physician who had to fill in the death certificate. We grouped the disorders to cardiovascular or to cancer death according to the main causal disorder or the disorder that inferred death using the International Classification of Diseases, version 10 [27].

### Data analysis

The data analysis was performed in 3 steps after presenting baseline characteristics of the study participants (Table 1): First, we analyzed associations of alcohol abstinence and alcohol consumption with time to death and used alcohol abstainers as one group of study participants. Low to moderate alcohol consumers were taken as the reference group (Table 2). Second, we analyzed subgroups of the alcohol-abstinent respondents according to one or more criteria for lifetime alcohol or drug dependence or abuse fulfilled, alcohol risky drinking, having made efforts to cut down or stop drinking, tobacco smoking status, and self-rated health. Again, low to moderate alcohol consumers were taken as the reference group (Table 2). In a third step, we again analyzed the subgroups of alcohol-abstinent respondents but used low to moderate alcohol consumers who had never smoked as the reference group (Table 3). This is due to low to moderate alcohol consumers having been utilized as one entire group for comparisons in research before [4]. In addition, we provided data analysis of subgroups. The alcohol abstainers are analyzed with reference to the low to moderate alcohol consumers for each subgroup (Table 4).

**Table 1:** Baseline characteristics of the study participants by baseline levels of alcohol consumption.

Characteristic		Alcohol consumption					
	Total n (column %)	Abstinent (AUDIT-C = 0) n (column %)	Low to moderate (AUDIT-C = 1–3) n (column %)	Moderate to high (AUDIT-C = 4) n (column %)	High (AUDIT-C = 5) n (column %)	Very high (AUDIT-C = 6–7) n (column %)	Extremely high (AUDIT-C = 8–12) n (column %)
Total	4,028 (100.0)	447 (100.00)	2,203 (100.00)	674 (100.00)	383 (100.00)	228 (100.00)	93 (100.00)
Demographic variables							
Females	2,006 (49.80)	248 (55.48)	1,368 (62.10)	250 (37.09)	89 (23.24)	37 (16.23)	14 (15.05)
Age groups							
18–39	1,851 (45.95)	161 (36.02)	1,040 (47.21)	330 (48.96)	174 (45.43)	104 (45.61)	42 (45.16)
40–49	847 (21.03)	85 (19.02)	435 (19.75)	156 (23.15)	95 (24.80)	52 (22.81)	24 (25.81)
50–64	1,330 (33.02)	201 (44.97)	728 (33.05)	188 (27.89)	114 (29.77)	72 (31.58)	27 (29.03)
School education							
<10 years	1,927 (47.84)	290 (64.88)	1,063 (48.25)	270 (40.06)	151 (39.43)	98 (42.98)	55 (59.14)
10–11 years	1,471 (36.52)	123 (27.52)	811 (36.81)	283 (41.99)	139 (36.29)	89 (39.04)	26 (27.96)
>11 years	630 (15.64)	34 (7.61)	329 (14.93)	121 (17.95)	93 (24.28)	41 (17.98)	12 (12.90)
Smoking status							
Never smoker	676 (16.78)	123 (27.52)	449 (20.38)	66 (9.79)	25 (6.53)	10 (4.39)	3 (3.23)
Ever less than daily	920 (22.84)	68 (15.21)	594 (26.96)	142 (21.07)	73 (19.06)	31 (13.60)	12 (12.90)
Former daily	839 (20.83)	85 (19.02)	407 (18.47)	171 (25.37)	99 (25.85)	61 (26.75)	16 (17.20)
Current daily <20 cpd	485 (12.04)	54 (12.08)	267 (12.12)	80 (11.87)	45 (11.75)	32 (14.04)	7 (7.53)
Current daily ≥20 cpd	1,108 (27.51)	117 (26.17)	486 (22.06)	215 (31.90)	141 (36.81)	94 (41.23)	55 (59.14)
Self-rated health							
Very good to excellent	1,427 (35.43)	111 (24.83)	797 (36.18)	265 (39.32)	145 (37.86)	81 (35.53)	28 (30.11)
Good	1,926 (47.82)	214 (47.87)	1,048 (47.57)	325 (48.22)	184 (48.04)	113 (49.56)	42 (45.16)
Fair to poor	675 (16.76)	122 (27.29)	358 (16.25)	84 (12.46)	54 (14.10)	34 (14.91)	23 (24.73)

Alcohol consumption: last 12 months prior to the interview at baseline. n number of study participants at baseline who had vital status information at follow-up. AUDIT-C, value range 0–12. The *p*-values of the Pearson chi-squared tests are < .001 for all characteristics shown.

AUDIT-C, Alcohol Use Disorder Identification Test-Consumption; cpd, cigarettes per day.

**Table 2:** Baseline alcohol abstinence subgroups, alcohol consumption, and deceased study participants 20 years after baseline, Cox proportional hazards model and logistic regression analysis.

Baseline	Total mortality				Cardiovascular mortality				Cancer mortality			
Alcohol abstinence, consumption	N	Deceased	Unadjusted	Adjusted for age and sex	N	Deceased	Unadjusted	Adjusted for age and sex	N	Deceased	Unadjusted	Adjusted for age and sex
		n (%)	HR (CI)	HR (CI)		n (%)	OR (CI)	OR (CI)		n (%)		OR (CI)
Alcohol abstainers all												
Alcohol abstinent (AUDIT-C = 0)	447	119 (26.62)	2.60 (2.09–3.24)	2.06 (1.65–2.56)	389	61 (15.68)	3.85 (2.78–5.33)	2.88 (2.07–3.99)	370	42 (11.35)	1.69 (1.18–2.40)	1.69 (1.18–2.40)
Alcohol abstainer subgroups												
Former alcohol or drug dependence or abuse (subgroup 2)	84	32 (38.10)	4.02 (2.78–5.82)	2.44 (1.68–3.56)	64	12 (18.75)	5.14 (2.65–9.98)	2.74 (1.33–5.65)	67	15 (22.39)	4.81 (2.63–8.79)	3.39 (1.76–6.53)
Former alcohol risk drinking (subgroup 3)	14	6 (42.86)	4.82 (2.14–10.82)	2.77 (1.23–6.25)	12	4 (33.33)	11.15 (3.29–37.71)	6.48 (1.66–25.29)	10	2 (20.00)	4.17 (0.88–19.84)	2.57 (0.50–13.22)
Tried to cut down or to stop drinking (subgroup 4)	16	6 (37.50)	4.09 (1.82–9.19)	2.41 (1.07–5.44)	14	4 (28.75)	8.92 (2.74–28.98)	5.26 (1.35–20.53)	10	0 (0.00)	-	-
Current daily smoker 20 or more cigarettes per day (subgroup 5)	76	21 (27.63)	2.75 (1.76–4.29)	3.97 (2.54–6.22)	63	8 (12.70)	3.24 (1.50–7.01)	6.42 (2.77–14.88)	64	9 (14.06)	2.73 (1.32–5.65)	4.21 (1.94–9.17)
Current daily smoker 19 or less cigarettes per day (subgroup 6)	39	8 (20.51)	1.93 (0.96–3.91)	2.78 (1.37–5.62)	35	4 (11.43)	2.88 (0.99–8.33)	4.24 (1.26 14.25)	34	3 (8.82)	1.61 (0.49–5.35)	2.71 (0.75–9.72)
Former daily smoker (subgroup 7)	46	14 (30.43)	3.00 (1.75–5.15)	1.76 (1.03–3.02)	43	11 (25.58)	7.66 (3.74–15.70)	4.16 (1.85–9.34)	34	2 (5.88)	1.04 (0.25–4.40)	0.71 (0.16–3.10)
Health fair to poor (subgroup 8)	47	18 (38.30)	4.02 (2.49–6.49)	2.22 (1.37–3.59)	42	13 (30.95)	9.99 (5.02–19.88)	5.15 (2.46–10.77)	33	4 (12.12)	2.30 (0.80–6.65)	1.10 (0.37–3.24)
Health excellent, very good or good (subgroup 1)	125	14 (11.20)	0.99 (0.58–1.69)	0.88 (0.51–1.51)	116	5 (4.31)	1.00 (0.40–2.52)	0.88 (0.34–2.30)	118	7 (5.93)	1.05 (0.48–2.31)	0.89 (0.39–2.00)
Alcohol consumers												
Alcohol consumption low to moderate (AUDIT-C = 1–3)	2,203	248 (11.26)	1.00 [Reference]	1.00 [Reference]	2,073	89 (4.29)	1.00 [Reference]	1.00 [Reference]	2,103	119 (5.66)	1.00 [Reference]	1.00 [Reference]
Alcohol consumption moderate to high (AUDIT-C = 4)	674	81 (12.02)	1.07 (0.83–1.37)	1.06 (0.82–1.37)	634	29 (4.57)	1.07 (0.70–1.64)	1.04 (0.66–1.63)	640	35 (5.47)	0.96 (0.65–1.42)	1.06 (0.71–1.59)
Alcohol consumption high (AUDIT-C = 5)	383	58 (15.14)	1.37 (1.03–1.82)	1.26 (0.94–1.69)	350	22 (6.29)	1.50 (0.92–2.42)	1.33 (0.79–2.24)	350	22 (6.29)	1.12 (0.70–1.79)	1.16 (0.70–1.92)
Alcohol consumption very high (AUDIT-C = 6–7)	228	39 (17.11)	1.58 (1.13–2.22)	1.45 (1.02–2.05)	210	18 (8.57)	2.09 (1.23–3.54)	1.89 (1.07–3.34)	207	15 (7.25)	1.30 (0.75–2.27)	1.38 (0.76–2.49)
Alcohol consumption extremely high (AUDIT-C = 8–12)	93	28 (30.11)	3.03 (2.05–4.48)	3.02 (2.03–4.51)	80	15 (18.75)	5.14 (2.82–9.38)	6.26 (3.18–12.31)	73	8 (10.96)	2.05 (0.96–4.38)	2.42 (1.07–5.44)

Study participants with baseline and vital status data: 4,028. N number of persons at baseline. n number of persons who had been deceased. % proportion of deceased among the persons at baseline who had vital status information at follow-up. Cox proportional hazards model for total mortality. The Cox proportional hazards assumption according to the Schoenfeld criterion is fulfilled. Logistic regression analysis if the proportional hazards assumption was not fulfilled. Alcohol abstinent: last 12 months prior to the interview at baseline. Subgroup 1: estimated their health as good to excellent and had no criteria fulfilled for alcohol or drug dependence or abuse, had no alcohol risk drinking, had not tried to cut down or to stop alcohol drinking, and had never been daily smokers. Subgroup 2: ever had one or more criteria for an alcohol or drug dependence or abuse fulfilled in life. Subgroup 3: had none of the risk factors of subgroup 2 but had practiced alcohol risk drinking. Subgroup 4: had none of the risk factors of subgroups 2–3 but had tried to cut down or to stop alcohol drinking. Subgroup 5: had none of the risk factors of subgroups 2–4 but were current daily smokers of 20 or more cigarettes per day. Subgroup 6: had none of the risk factors of subgroups 2–5 but were current daily smokers of 19 or less cigarettes per day. Subgroup 7: had none of the risk factors of subgroups 2–6 but were former daily smokers. Subgroup 8: had none of the risk factors of subgroups 2–7 but disclosed fair to poor health. AUDIT-C, value range 0–12.–no death cases.

AUDIT-C, Alcohol Use Disorder Identification Test-Consumption; CI, confidence interval; HR, hazard ratio; OR, odds ratio.

**Table 3:** Baseline alcohol use, tobacco smoking, self-rated health, and deceased study participants 20 years after baseline, and time to death, Cox proportional hazards model, and logistic regression analysis.

Baseline	Total mortality				Cardiovascular mortality				Cancer mortality			
Alcohol abstinence, consumption	N	Deceased	Unadjusted	Adjusted for age and sex	N	Deceased	Unadjusted	Adjusted for age and sex	N	Deceased	Unadjusted	Adjusted for age and sex
	4,028	n (%)	HR (CI)	HR (CI)	3,736	n (%)	OR (CI)	OR (CI)	3,743	n (%)	OR (CI)	OR (CI)
Alcohol abstainer subgroups												
Alcohol abstinent, former alcohol or drug dependence or abuse (subgroup 2)	84	32 (38.10)	5.02 (3.16–7.97)	3.35 (2.08–5.39)	64	12 (18.75)	7.98 (3.41–18.68)	4.82 (1.92–12.12)	67	15 (22.39)	5.44 (2.66–11.14)	4.77 (2.18–10.46)
Alcohol abstinent, former alcohol risk drinking (subgroup 3)	14	6 (42.86)	6.01 (2.55–14.15)	3.78 (1.60–8.97)	12	4 (33.33)	17.29 (4.57–65.41)	11.25 (2.57–49.15)	10	2 (20.00)	4.72 (0.94–23.54)	3.53 (0.65–19.24)
Alcohol abstinent, tried to cut down or to stop drinking (subgroup 4)	16	6 (37.50)	5.10 (2.16–12.01)	3.25 (1.37–7.68)	14	4 (28.57)	13.83 (3.79–50.44)	9.08 (2.06–40.02)	10	0 (0.00)	-	-
Alcohol abstinent, current daily smoker 20 or more cigarettes per day (subgroup 5)	76	21 (27.63)	3.43 (2.03–5.80)	5.59 (3.29–9.49)	63	8 (12.70)	5.03 (1.97–12.85)	11.63 (4.22–32.01)	64	9 (14.06)	3.09 (1.35–7.04)	6.17 (2.54–15.00)
Alcohol abstinent, current daily smoker 19 or less cigarettes per day (subgroup 6)	39	8 (20.51)	2.41 (1.13–5.15)	3.84 (1.80–8.19)	35	4 (11.43)	4.46 (1.36–14.65)	7.39 (1.93–28.28)	34	3 (8.82)	1.83 (0.52–6.44)	3.87 (0.997–14.99)
Alcohol abstinent, former daily smoker (subgroup 7)	46	14 (30.43)	3.75 (2.04–6.87)	2.34 (1.27–4.31)	43	11 (25.58)	11.89 (4.86–29.06)	7.01 (2.63–18.70)	34	2 (5.88)	1.18 (0.27–5.24)	0.95 (0.21–4.39)
Alcohol abstinent, health fair to poor (subgroup 8)	47	18 (38.30)	5.02 (2.88–8.73)	2.92 (1.68–5.09)	42	13 (30.95)	15.50 (6.49–37.01)	8.59 (3.44–21.47)	33	4 (12.12)	2.60 (0.84–8.05)	1.42 (0.45–4.52)
Alcohol abstinent, health excellent, very good or good (subgroup 1)	125	14 (11.20)	1.23 (0.67–2.26)	1.18 (0.64–2.16)	116	5 (4.31)	1.56 (0.54–4.51)	1.48 (0.49–4.46)	118	7 (5.93)	1.19 (0.50–2.86)	1.18 (0.47–2.93)
Alcohol consumers												
Alcohol consumption low to moderate (AUDIT-C = 1–3), never smoker	449	41 (9.13)	1.00 [Reference]	1.00 [Reference]	427	12 (2.81)	1.00 [Reference]	1.00 [Reference]	437	22 (5.03)	1.00 [Reference]	1.00 [Reference]
Alcohol consumption low to moderate (AUDIT-C = 1–3), ever less than daily or former daily smoker	1,001	97 (9.69)	1.06 (0.74–1.53)	1.04 (0.72–1.51)	959	44 (4.59)	1.66 (0.87–3.18)	1.56 (0.80–3.06)	956	41 (4.29)	0.85 (0.50–1.44)	0.90 (0.52–1.57)
Alcohol consumption low to moderate (AUDIT-C = 1–3), current daily smoker 19 or less cigarettes per day	267	37 (13.86)	1.55 (0.99–2.41)	2.10 (1.34–3.28)	250	12 (4.80)	1.74 (0.77–3.94)	2.71 (1.15–6.36)	255	17 (6.67)	1.35 (0.70–2.59)	2.18 (1.10–4.31)
Alcohol consumption low to moderate (AUDIT-C = 1–3), current daily smoker 20 or more cigarettes per day	486	73 (15.02)	1.71 (1.16–2.50)	2.40 (1.63–3.54)	437	21 (4.81)	1.75 (0.85–3.59)	2.85 (1.33–6.09)	455	39 (8.57)	1.77 (1.03–3.03)	3.06 (1.72–5.46)
Alcohol consumption moderate to high or high (AUDIT-C = 4–5), never smoker	91	8 (8.79)	0.97 (0.46–2.07)	0.87 (0.41–1.86)	85	2 (2.35)	0.83 (0.18–3.79)	0.70 (0.15–3.28)	88	5 (5.68)	1.14 (0.42–3.09)	1.07 (0.38–3.01)
Alcohol consumption moderate to high or high (AUDIT-C = 4–5), ever less than daily or former daily smoker	485	55 (11.34)	1.25 (0.83–1.87)	1.15 (0.76–1.74)	461	22 (4.77)	1.73 (0.85–3.55)	1.49 (0.70–3.19)	462	23 (4.98)	0.99 (0.54–1.80)	1.09 (0.58–2.06)
Alcohol consumption very high (AUDIT-C = 4–5), current daily smoker 19 or less cigarettes per day	125	12 (9.60)	1.06 (0.56–2.01)	1.51 (0.79–2.90)	117	2 (1.71)	0.60 (0.13–2.73)	0.97 (0.20–4.60)	120	5 (4.17)	0.82 (0.30–2.21)	1.38 (0.49–3.89)
Alcohol consumption very high (AUDIT-C = 4–5), current daily smoker 20 or more cigarettes per day	356	64 (17.98)	2.05 (1.39–3.04)	2.81 (1.87–4.23)	321	25 (7.79)	2.92 (1.44–5.91)	4.41 (2.05–9.48)	320	24 (7.50)	1.53 (0.84–2.78)	3.07 (1.59–5.90)
Alcohol consumption very high (AUDIT-C = 6–7), never, ever less than daily or former daily smoker	102	8 (7.84)	0.85 (0.40–1.81)	0.77 (0.36–1.65)	99	3 (3.03)	1.08 (0.30–3.90)	0.90 (0.24–3.38)	100	4 (4.00)	0.79 (0.26–2.33)	0.88 (0.29–2.73)
Alcohol consumption very high (AUDIT-C = 6–7), current daily smoker 19 or less cigarettes per day	32	5 (15.63)	1.79 (0.71–4.53)	2.14 (0.84–5.45)	28	1 (3.57)	1.28 (0.16–10.22)	1.80 (0.20–16.30)	29	2 (6.90)	1.40 (0.31–6.26)	2.08 (0.41–10.49)
Alcohol consumption very high (AUDIT-C = 6–7), current daily smoker 20 or more cigarettes per day	94	26 (27.66)	3.44 (2.10–5.62)	4.06 (2.45–6.74)	83	14 (16.87)	7.02 (3.12–15.81)	9.18 (3.76–22.46)	78	9 (11.54)	2.46 (1.09–5.57)	4.18 (1.70–10.28)
Alcohol consumption extremely high (AUDIT-C = 8–12), never ever less than daily or former daily smoker	31	8 (25.81)	3.25 (1.52–6.93)	2.92 (1.36–6.29)	27	4 (14.81)	6.01(1.80–20.11)	6.05 (1.65–22.20)	25	2 (8.00)	1.64 (0.36–7.40)	1.63 (0.34–7.86)
Alcohol consumption extremely high (AUDIT-C = 8–12), current daily smoker	62	20 (32.26)	4.04 (2.37–6.90)	5.27 (3.04–9.14)	53	11 (20.75)	9.06 (3.77–21.78)	16.60 (6.16–44.68)	48	6 (12.50)	2.69 (1.04–7.02)	5.44 (1.90–15.54)

Study participants with baseline and vital status data: 4,028. N number of persons at baseline. n number of persons who had been deceased. % proportion of deceased among the persons at baseline who had vital status information at follow-up. Cox proportional hazards model for total mortality. The Cox proportional hazards assumption according to the Schoenfeld criterion is fulfilled. Logistic regression analysis if the proportional hazards assumption was not met. Alcohol abstinent: last 12 months prior to the interview at baseline. Subgroup 1: estimated their health as good to excellent and had no criteria fulfilled for alcohol or drug dependence or abuse, had no alcohol risk drinking, had not tried to cut down or to stop alcohol drinking, and had been never or ever less than daily but not daily smokers. Subgroup 2: ever had one or more criteria for an alcohol or drug dependence or abuse fulfilled in life and were never, ever less than daily, former daily, or current daily smoker. Subgroup 3: had none of the risk factors of subgroup 2 but had practiced alcohol risk drinking and were never, ever less than daily, former daily, or current daily smoker. Subgroup 4: had none of the risk factors of subgroups 2–3 but had tried to cut down or to stop alcohol drinking and were never, ever less than daily, former daily, or current daily smoker. Subgroup 5: had none of the risk factors of subgroups 2–4 but were current daily smokers of 20 or more cigarettes per day. Subgroup 6: had none of the risk factors of subgroups 2–5 but were current daily smokers of 19 or less cigarettes per day. Subgroup 7: had none of the risk factors of subgroups 2–6 but were former daily smokers. Subgroup 8: had none of the risk factors of subgroups 2–7 but disclosed fair to poor health and were never or ever less than daily smoker. AUDIT-C, value range 0–12.—no death cases.

AUDIT-C, Alcohol Use Disorder Identification Test-Consumption; CI, confidence interval; HR, hazard ratio; OR, odds ratio.

**Table 4:** Baseline alcohol consumption low to moderate versus alcohol abstinence in subgroups, deceased study participants 20 years after baseline, and time to death, Cox proportional hazards model.

Subgroups	Total mortality							
	Baseline alcohol consumption low to moderate			Baseline alcohol abstinent				Pearson chi-squared significance*
	N	Deceased		N	Deceased	Unadjusted	Adjusted for age and sex	
		n (%)	HR		n (%)	HR (CI)	HR (CI)	
Former alcohol or drug dependence or abuse (subgroup 2)	167	15 (8.98)	1.00 [Reference]	85	33 (38.82)	5.15 (2.79–9.49)	3.12 (1.64–5.93)	< .001
Former alcohol risk drinking (subgroup 3)	69	8 (11.59)	1.00 [Reference]	14	6 (42.86)	4.69 (1.62–13.58)	4.02 (1.28–12.61)	< .01
Had tried to cut down or to stop drinking (subgroup 4)	77	13 (16.88)	1.00 [Reference]	15	5 (33.33)	2.18 (0.78–6.13)	1.40 (0.49–3.99)	= .142
Current daily smoker 20 or more cigarettes per day (subgroup 5)	392	58 (14.80)	1.00 [Reference]	76	21 (27.63)	2.03 (1.23–3.35)	2.29 (1.38–3.80)	< .01
Current daily smoker 19 or less cigarettes per day (subgroup 6)	238	33 (13.87)	1.00 [Reference]	39	8 (20.51)	1.56 (0.72–3.78)	1.96 (0.90–4.27)	= .279
Former daily smoker (subgroup 7)	317	38 (11.99)	1.00 [Reference]	45	14 (31.11)	2.83 (1.53–5.23)	1.96 (1.05–3.67)	< .01
Ever less than daily smoker (subgroup 7a)	527	45 (8.54)	1.00 [Reference]	62	6 (9.68)	1.16 (0.49–2.71)	1.00 (0.43–2.35)	= .763
Health fair to poor (subgroup 8)	80	12 (15.00)	1.00 [Reference]	31	12 (38.71)	3.12 (1.40–6.95)	3.00 (1.34–6.71)	< .01
Health excellent, very good, or good (subgroup 1)	330	26 (7.88)	1.00 [Reference]	79	14 (17.72)	2.34 (1.22–4.48)	1.88 (0.97–3.62)	< .01

Cox proportional hazards model. Study participants with baseline and vital status data. Alcohol consumption low to moderate: AUDIT-C 1–3. Alcohol abstinent: last 12 months alcohol abstinent last 12 months prior to the baseline interview according to AUDIT-C. N number of persons at baseline. n number of persons who had been deceased. % proportion of deceased among the persons at baseline who had vital status information at follow-up. The Cox proportional hazards assumption according to the Schoenfeld criterion is fulfilled except sex in subgroup 1 in model 2. Subgroup 1: estimated their health as good to excellent and had no criteria fulfilled for alcohol or drug dependence or abuse, had no alcohol risk drinking, had not tried to cut down or to stop alcohol drinking, and had never smoked in life before. Subgroup 2: ever had one or more criteria for an alcohol or drug dependence or abuse fulfilled in life and were never, ever less than daily, former daily, or current daily smoker. Subgroup 3: had none of the risk factors of subgroup 2 but had practiced alcohol risk drinking and were never, ever less than daily, former daily, or current daily smoker. Subgroup 4: had none of the risk factors of subgroups 2–3 but had tried to cut down or to stop alcohol drinking and were never, ever less than daily, former daily, or current daily smoker. Subgroup 5: had none of the risk factors of subgroups 2–4 but were current daily smokers of 20 or more cigarettes per day. Subgroup 6: had none of the risk factors of subgroups 2–5 but were current daily smokers of 19 or less cigarettes per day. Subgroup 7: had none of the risk factors of subgroups 2–6 but were former daily smokers. Subgroup 7a: had none of the risk factors of subgroups 2–6 but were ever less than daily smokers. Subgroup 8: had none of the risk factors of subgroups 2–7a but disclosed fair to poor health and had never smoked in life before.

AUDIT-C, Alcohol Use Disorder Identification Test-Consumption; CI, confidence interval; HR, hazard ratio adjusted for age and sex.

The data analysis had been planned in advance of the data gathering for the mortality follow-up except the data analysis of subgroups presented in Table 4. It was evolved from the review process of this paper.

The dependent variable was time to death beginning at the day of the baseline interview. For all individuals who were still alive at the date of the mortality follow-up, the time was truncated at this date. The Pearson chi-squared test was used for proportions. A *p*-value < .05 was estimated as significant. We calculated Cox proportional hazards models and present the hazard ratios (HRs) with 95% confidence intervals (CIs). We tested the proportional hazards assumption using Schoenfeld residuals and present models that fulfilled the proportional hazards assumption [28,29]. We safeguarded that the minimum number of outcome events (death cases) per predictor variable was 5 or higher [30]. If the proportional hazards assumption had not been met or the number of death cases was less than 5, logistic regression analysis was used. In this case, odds ratios (ORs) and 95% CIs are given. The data for unadjusted models and models adjusted for age and sex are presented.

Missing values in the AUDIT-C were replaced by the mean of the AUDIT-C considering sex and age group: 8 missing values in the first, 23 missing values in the second, and 4 missing values in the third AUDIT-C item. Together, the missing values represent 0.3% of all AUDIT-C item responses. To visualize survival time, we plotted the estimated distributions for each group. All data were analyzed using Stata 15.1 [31]. This study is reported according to the Strengthening the Reporting of Observational Studies in Epidemiology (STROBE) guideline (S1 STROBE Checklist). Although the study did not have a formal prospective statistical analysis plan, all analyses were planned except for the analysis of the subgroups presented in Table 4. These analyses were added during the peer review process. The study did not have a protocol. The baseline data collection for this study did not require ethical approval. For the follow-up mortality data collection, we received a statement from the ethics committee of the University Medicine Greifswald stating that it had no objections (Ethics statement number: BB044/13, 02.05.2013).

## Results

Among the 4,028 study participants at baseline, 447 (11.10%) had not drunk any alcohol in the past 12 months prior to the baseline interview (Table 1). Among these abstainers, 248 (55.48%) were female, 201 (44.97%) at age 50 to 64, and 122 (27.29%) self-rated their health in general as fair to poor. Among low to moderate alcohol consumers, 1,368 (62.10%) were female, 728 (33.05%) at age 50 to 64, and 358 (16.25%) who self-rated their health in general as fair to poor.

Among the 447 study participants who had abstained from alcohol at baseline, 405 (90.60%) were former alcohol consumers and 42 (9.40%) lifetime abstainers. Among them, 6 (14.29%) had been deceased. The proportion was not significantly higher than among the low to moderate alcohol consumers (Pearson chi-squared = 0.38; *p* = .54). The HR for lifetime abstainers compared to low to moderate alcohol consumers after adjustment for age, sex, and tobacco smoking status was 1.64 (0.72 to 3.77).

Among the 447 alcohol-abstinent study participants at baseline, 119 (26.62%) had been deceased, whereas among the 2,203 study participants with low to moderate alcohol consumption during the last 12 months prior to the baseline interview, 248 (11.26%) died (Pearson chi-squared *p* < .001). Increased HRs were found for the study participants who had been alcohol abstinent during the last 12 months and for those with high to extremely high alcohol consumption compared to those with low to moderate alcohol consumption after adjustment for age and sex. This was true for total, cardiovascular, and cancer mortality after adjustment for age and sex (Table 2). The HR for the AUDIT-C for all study participants who had abstained from alcohol during the past 12 months prior to the baseline interview compared to the low to moderate alcohol consumers was 2.06 (1.65 to 2.56) for total mortality and 2.88 (2.07 to 3.99) for cardiovascular mortality after adjustment for age and sex (Table 2). The HR for a 1-point increase of the AUDIT-C among all who had consumed alcohol (AUDIT-C range: 1 to 12) in the past 12 months prior to the baseline interview was 1.11 (1.06 to 1.17).

Among all alcohol-abstinent study participants at baseline, we found 8 distinct subgroups according to risk factors. The 125 respondents of subgroup 1 (27.96% of the alcohol abstainers) had estimated their health as good to excellent and had no criteria for alcohol or drug dependence or abuse fulfilled, had no alcohol risky drinking, had not tried to cut down or to stop alcohol drinking, and had never been daily smokers in their life before. Subgroups 2 to 8 included 322 (72.04%) of the alcohol abstainers at baseline with one or more of the risk factors analyzed. Among them, 114 (35.40%) had an alcohol use disorder or risky alcohol consumption in their history. Another 161 (50.00%) did not have such an alcohol-related risk but were daily smokers. Subgroup 2 (84 individuals, 18.79% of the alcohol abstainers) ever had one or more criteria for an alcohol or drug dependence or abuse fulfilled in life before. Among these, we found 44 former alcohol-dependent study participants. Subgroup 3 (14 individuals, 3.13% of the alcohol abstainers) had none of the disorders of subgroup 2 but had practiced alcohol risky drinking in the time before 1 year prior to the baseline interview. Subgroup 4 (16 individuals, 3.58% of the alcohol abstainers) had none of the risk factors of subgroups 2 to 3 but had agreed with “I try to cut down on drinking alcohol or to stop drinking alcohol at all.” Subgroup 5 (76 individuals, 17.00% of the alcohol abstainers) had none of the risk factors of subgroups 2 to 4 but were current daily smokers of 20 or more cigarettes per day. Subgroup 6 (39 individuals, 8.72% of the alcohol abstainers) had none of the risk factors of subgroups 2 to 5 but were current daily smokers of 19 or less cigarettes per day. Subgroup 7 (46 individuals, 10.29% of the alcohol abstainers) had none of the risk factors of subgroups 2 to 6 but were former daily smokers. Subgroup 8 (47 individuals, 10.51% of the alcohol abstainers) had none of the risk factors of subgroups 2 to 7 but disclosed fair to poor health.

**Fig 1:**
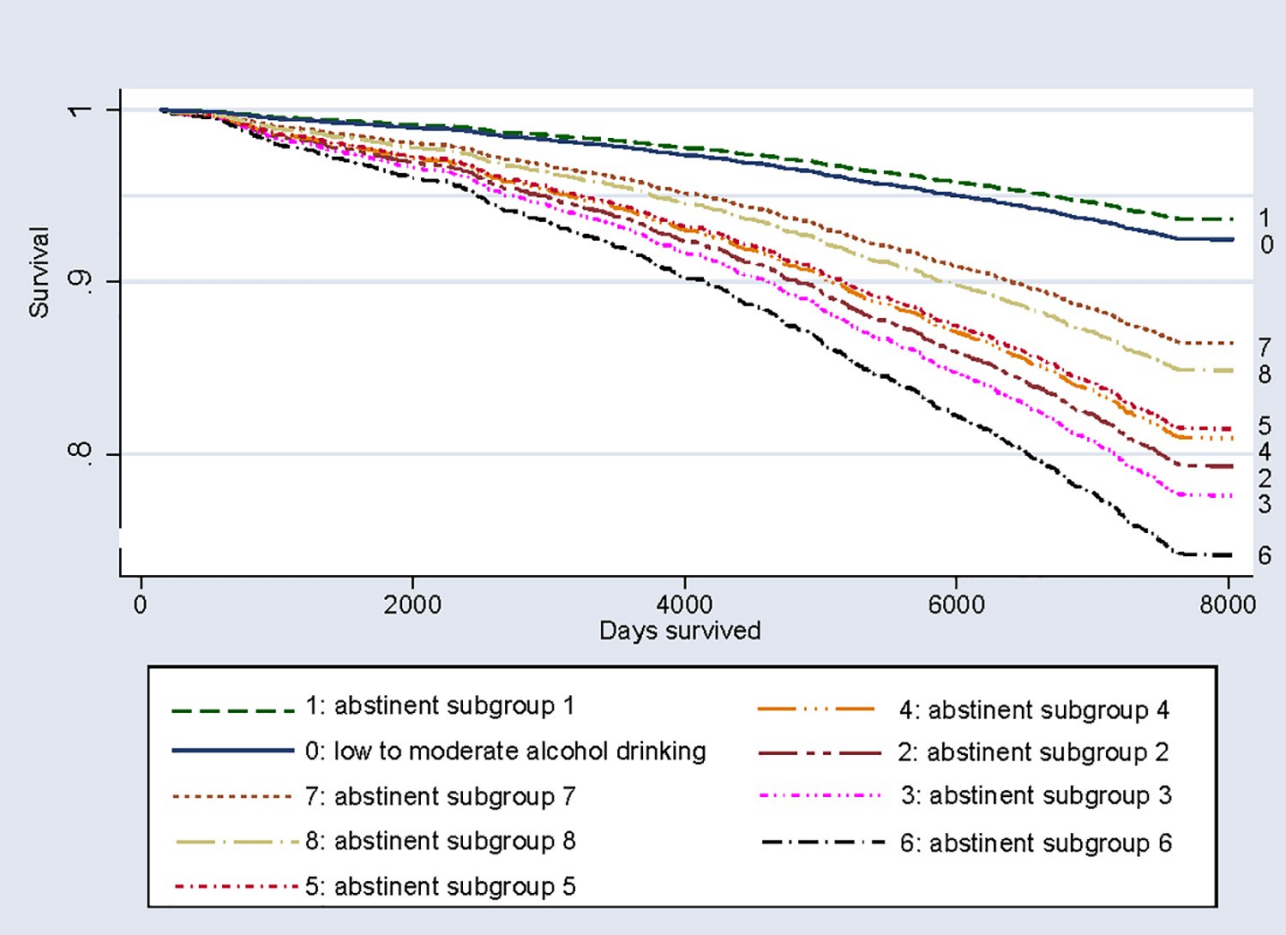
Survival of baseline alcohol abstainers, low to moderate drinkers, Cox proportional hazards regression.

For all study participants with alcohol abstinence during the last 12 months prior to the baseline interview except subgroup 1, increased HRs for total mortality and increased ORs for cardiovascular mortality were revealed by the data after adjustment for age and sex (Table 2 and Fig 1). For cancer mortality, increased ORs existed for those with former alcohol or drug use disorders and for current daily smokers of 20 or more cigarettes per day. Data for respondents of subgroup 1 did not show a statistically significant difference in mortality risk compared with low to moderate alcohol consumers. This was found for total, for cardiovascular, and for cancer mortality. Fig 1 illustrates findings according to the alcohol-abstinent study participants. Survival of the reference group and subgroup 1 was similar, but survival rate was lower for abstinent participants who fulfilled one or more criteria for alcohol or drug dependence or abuse, had a history of alcohol risky drinking, had ever attempted to cut down or stop drinking alcohol, had ever been daily smoker, or had reported fair to poor health.

**Fig 2:**
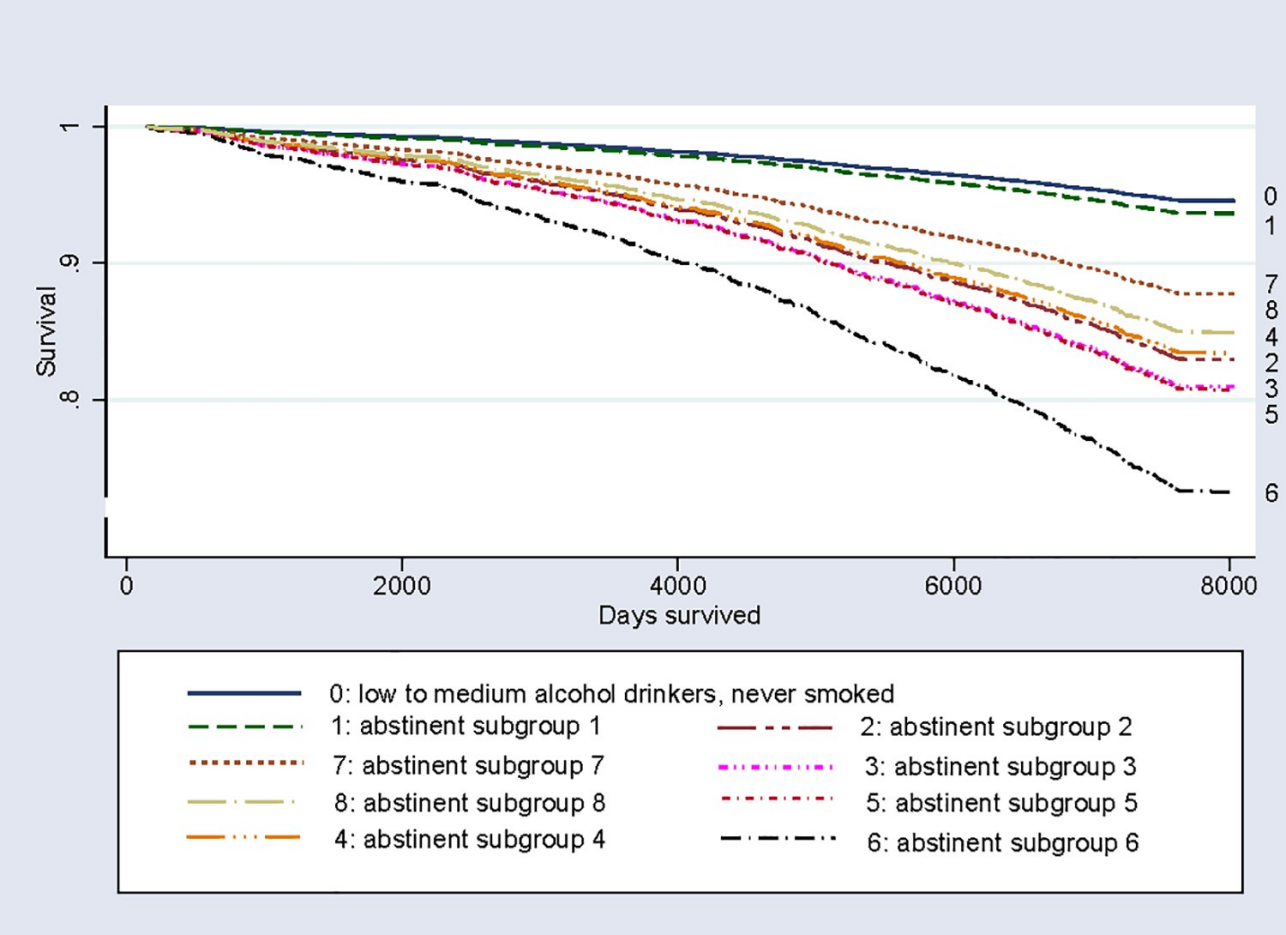
Survival of baseline alcohol abstainers, low to moderate drinkers who never smoked, Cox proportional hazards regression.

In a next step, we took persons with low to moderate alcohol drinking and never having smoked in life before as the reference group. Increased and particularly high HRs and ORs for total and for cardiovascular mortality were found among all alcohol abstinent subgroups except subgroup 1 (Table 3). According to cancer mortality, increased ORs were found for abstinent participants with criteria for former alcohol or drug dependence or abuse fulfilled and for current daily smokers of 20 or more cigarettes per day. The survival curves show the reference group and subgroup 1 being close together and the other subgroups of abstainers having lower survival (Fig 2).

Taking single subgroups of low to moderate drinkers as the reference groups, our data revealed that the alcohol abstainers who had good to excellent health, never smoked daily, and no alcohol risk factors did not have significantly higher HRs for time to death than the same subgroup among the low to moderate alcohol consumers after adjustment for age and sex (Table 4). The data revealed higher proportions of deceased persons among the abstainers for all subgroups (Pearson chi-squared *p* < .001). Abstainers who ever had one or more criteria for an alcohol or drug use disorder or abuse fulfilled in their life before had an HR 3.12 (1.64 to 5.93) compared to current low to moderate drinkers with the same risk factors as the reference group. Alcohol abstainers who had disclosed former alcohol risky drinking, had an HR 4.02 (1.28 to 12.61) after adjustment for age and sex. Abstainers who were current smokers of 20 or more cigarettes per day or former daily smokers or were of fair to poor health had higher HRs for time to death than the equivalent low to moderate alcohol consumers.

## Discussion

There are 3 main findings of this adult general population study. First, among the abstainers at baseline, 90.60% had a history of alcohol consumption. Second, the majority of alcohol abstainers at baseline had risk factors for early death: past risky alcohol consumption, tobacco smoking, and self-rated health being less than good seem to predict early death. Third, alcohol abstainers with no obvious history of alcohol-related risks or daily tobacco smoking who self-rated their health as good, very good, or excellent had a life expectancy similar to that of low to moderate alcohol drinkers.

Among all abstainers, 90.60% were former alcohol consumers. For the study participants who had claimed to never having drunk alcohol in their life before, no increased HRs compared to low to moderate alcohol consumers were found after adjustment for age and sex. The finding supports evidence before [4,6]. These study participants were just 1% of the baseline sample what also corresponds to evidence shown before [11]. These findings speak against health protective effects of alcohol consumption. However, it has to be kept in mind that after adjustment for age and sex, the HR was not statistically significant but larger than 1. It cannot be precluded that in larger samples, the HR might become significant.

Among all alcohol-abstinent study participants at baseline, 72.04% had one or more of the factors analyzed that are known to be a risk of early death. The risks are similar to those of study participants with the highest alcohol consumption. Among the alcohol abstainers at baseline, the HRs for time to death might largely be explained by 3 risk factors: former alcohol- or drug-related behavior (having fulfilled one or more criteria for former alcohol or drug use disorders or alcohol risky drinking or ever having tried to cut down or to stop alcohol consumption), by tobacco smoking, and by fair to poor health. Among the 322 abstinent study participants with one or more of the risk factors analyzed, 35.40% had an alcohol use disorder or risky alcohol consumption in their history. Another 50.00% did not have such an alcohol-related risk but were daily smokers. This finding suggests that alcohol consumption and tobacco smoking might explain the majority of health risks that have been disclosed by the abstinent persons with one or more risks for death. The data revealed that alcohol abstainers include subgroups with very high risks of death. When the low to moderate alcohol consumers who never smoked were taken as the reference group, the HRs of the alcohol-abstinent subgroups with any former alcohol-related risk or current daily smoking at baseline were consistently higher than 3 for total mortality. These findings suggest that former alcohol consumption and tobacco smoking should be considered when risks of death among current alcohol abstainers are estimated. This seems to be plausible also in the light of particularly high risks of death from the combination of alcohol drinking and tobacco smoking [13,14]. The use of self-rated health adds to inform potential causes of death among abstainers beyond former alcohol- or drug-related behaviors or tobacco smoking. The results are in line with those that have shown increased risks for total [16], cardiovascular [16,17], and cancer mortality [16] among people who disclose fair to poor health in their own view.

The data suggest that single subgroups of alcohol abstainers have higher HRs than low to moderate alcohol consumers who have the same risk factors. Abstainers who had fulfilled one or more criteria for an alcohol or drug use disorder, who had been former risky alcohol consumers, who were current daily smokers of 20 or more cigarettes per day, or who were former daily smokers and abstainers who had rated their health as fair to poor might be persons with higher burden of disease than the equivalent groups among low to moderate alcohol consumers. This result shows that the risk factors that we analyzed do not fully explain the increased likelihood of early death among abstainers. Residual confounding is likely. Different degrees of pathology may exist among the abstainers and among the low to moderate consumers. Due to a potentially higher burden of disease, the felt need to stay abstinent might be stronger among the alcohol abstainers than among low to moderate alcohol consumers. This is supported by the finding that the data did not reveal statistically increased HRs for abstainers with seemingly less severe risk factors: having tried to cut down or to stop alcohol consumption, having smoked 19 or less cigarettes per day, or having ever smoked in life but not daily. The magnitude of the HRs suggests that the increased mortality may not be explained by a potential health protective effect of low to moderate alcohol consumption. Instead, the subgroups among abstainers might include persons with health disorders that are insufficiently described by the risk factors that have been analyzed. These include the estimation of health in general, which has been shown to predict mortality [16]. However, we have not considered specific medical conditions. Medications, e. g., may require alcohol abstinence.

Alcohol abstainers at baseline who did not disclose a history of alcohol-related risks or daily tobacco smoking and who had self-rated their health as good, very good, or excellent clearly did not statistically differ from low to moderate drinkers in total. Also, the findings of the more specific comparisons of this group of alcohol abstainers with low to moderate alcohol consumers who had never smoked daily suggest that no statistical difference exists between alcohol abstainers and low to moderate consumers. This is true for total, for cardiovascular, and for cancer mortality. However, the comparison between alcohol abstainers and low to moderate consumers within the subgroup of study participants who had good, very good, or excellent health and no history of alcohol-related risks or daily tobacco smoking is close to revealing a significantly increased risk for the baseline abstainers. This finding shows that residual confounding is likely.

Our findings support a dose relation between alcohol drinking and time to death. Asking for alcohol consumption and using simple questions were sufficient to predict mortality 20 years later. Our data are in line with those from former studies that revealed a dose relation between quantity and frequency of alcohol consumption and risk of death [4,5,32].

Taken together, the results of our study speak against the assumption that alcohol drinking might have a protective effect on health or a potential to decrease hazards of time to death. First, the findings suggest that both lifetime alcohol abstainers and abstainers who had been former alcohol consumers but had none of the risk factors analyzed do not statistically differ from low to moderate alcohol consumers in time to death. Second, we found considerable numbers of persons who met criteria of former alcohol-related risk behavior, alcohol or drug dependence among the current abstainers. Third, a further alcohol-related risk is tobacco smoking. Its effects include that tobacco smoking may stimulate alcohol drinking and vice versa [12–14]. The results speak in favor of not to recommend any alcohol consumption for health reasons.

Strengths of this study are that a general adult population sample and standardized measures of alcohol consumption and alcohol use disorders have been used. Clear time frames of alcohol consumption were provided and potential reasons of abstinence analyzed. Limitations include the following: (1) The sample from the general adult population had been drawn in one region of one country only. Proportions of abstainers and their subgroups may be different in other countries. (2) Only tobacco smoking as a health risk behavior was used beyond alcohol drinking. However, smoking may interact with alcohol consumption most closely. We restricted our data analysis to variables that may be causal in the prediction of time to death and that are related to alcohol consumption. (3) We did not gather data about specific medical conditions among the abstainers. We used only self-statements from baseline and did not validate fair to poor health. However, there is evidence that self-rated health can be predictive of mortality. (4) Underreporting must be assumed for alcohol consumption. Also, the validity of self-statements about abstinence from alcohol is limited [33]. (5) This is an observational study and not sufficient to prove causality. However, we found clear-cut relations between risks of alcohol-abstinent participants and mortality that are in line with known risks of death. (6) The data for subgroups in Tables 3 and 4 include small numbers of individuals what added to wide CIs of the HRs.

## Conclusions

The results support the view that people in the general population who currently are abstinent from alcohol do not necessarily have a shorter survival time than the population with low to moderate alcohol consumption. Increased mortality risks among abstainers might largely be explained by previous alcohol or drug problems, risky drinking, daily smoking, and self-rated health as fair to poor. The findings speak against recommendations to drink alcohol for health reasons.

## Supporting information

S1 STROBE Checklist Checklist according to the Strengthening the Reporting of Obsrvationla Studie in Epidemiology (STROBE) guideline. (DOCX)

S1 Data Study data. (XLS)

## Abbreviations

AUDIT-C: Alcohol Use Disorder Identification Test-Consumption
CI: confidence interval
HR: hazard ratio
OR: odds ratio
